# A New Insight into the Roles of MiRNAs in Metabolic Syndrome

**DOI:** 10.1155/2018/7372636

**Published:** 2018-12-17

**Authors:** Yuxiang Huang, Yuxiang Yan, Weicheng Xv, Ge Qian, Chijian Li, Hequn Zou, Yongqiang Li

**Affiliations:** ^1^Department of Nephrology, Institute of Nephrology and Urology, The Third Affiliated Hospital of Southern Medical University, Guangzhou, China; ^2^Department of Epidemiology and Biostatistics, School of Public Health, Capital Medical University, Beijing, China

## Abstract

Metabolic syndrome (MetS), which includes several clinical components such as abdominal obesity, insulin resistance (IR), dyslipidemia, microalbuminuria, hypertension, proinflammatory state, and oxidative stress (OS), has become a global epidemic health issue contributing to a high risk of type 2 diabetes mellitus (T2DM). In recent years, microRNAs (miRNAs), used as noninvasive biomarkers for diagnosis and therapy, have aroused global interest in complex processes in health and diseases, including MetS and its components. MiRNAs can exist stably in serum, liver, skeletal muscle (SM), heart muscle, adipose tissue (AT), and *β*cells, because of their ability to escape the digestion of RNase. Here we first present an overall review on recent findings of the relationship between miRNAs and several main components of MetS, such as IR, obesity, diabetes, lipid metabolism, hypertension, hyperuricemia, and stress, to illustrate the targeting proteins or relevant pathways that are involved in the progress of MetS and also help us find promising novel diagnostic and therapeutic strategies.

## 1. Introduction

MetS, once identified as ‘IR syndrome', ‘syndrome X', ‘hypertriglyceridemic waist', and ‘the deadly quartet', was firstly globally defined as MetS in 1998 by the Diabetes Consultation Group of the World Health Organization. MetS is a combination of several characteristics, including obesity (especially abdominal adiposity), glucose intolerance, IR, dyslipidemia (including hypertriglyceridemia, increasing free fatty acid (FFA), and decreased HDL cholesterol (HDL-C)), microalbuminuria, hypertension, nonalcoholic fatty liver disease (NAFLD), polycystic ovarian syndrome (PCOS), the proinflammatory state, and oxidative stress, resulting in an increasing risk of T2DM, cardiovascular disease (CVD), fatty liver, and cancer [1–6]. Five risk factors with different critical points are used to identify individuals with the MetS: waist circumference, circulating levels of triglycerides and HDL cholesterol, fasting glucose, and blood pressure. IR also plays a pivotal role on the MetS [5, 7].

With these risk factors, it has been demonstrated clearly that the syndrome has become a global health challenge, which has a close correlation with increasing adiposity and sedentary lifestyles [8]. Patients with MetS would spend more than those without MetS with a 20% increase in cost if they suffered every additional component of MetS [4]. Alarmingly, in accordance with a survey conducted by Roy A. Wong-McClure et al, in 2015, more than 30% of the samples involved have MetS. Respectively, a meta-analysis with a total population of 226,653 Chinese subjects from mainland China shows that there were 24.5 % of the subjects suffering from the syndrome [9]. In female subjects, the frequency of MetS rose sharply with increasing age. In males, although the frequency of MetS was significantly higher in older subjects, it remained stable among older age groups (usually over 45 or 50 years old). In the younger age group, the prevalence of MetS was higher in males than females, but the pooled prevalence in women tended to be higher than that in men [9–11]. Individuals living in urban areas were more susceptible to the MetS [9]. The increasing morbidity of MetS has caused a rise in the rate of global morbidity and mortality due to CVD and T2DM. Though we can alleviate the syndrome through lifestyle improvement, a wholesome diet, and pharmacological treatment [12], early detection and diagnosis of the syndrome are of great importance, allowing for early therapeutic intervention [13].

In recent years, miRNAs have arisen global interest in complex processes in health and diseases, including MetS and its components, used as noninvasive biomarkers for diagnosis [13, 14]. MiRNAs were discovered in 1993. Lin-4 and let-7 were the first well-characterized “small” RNAs that were found to control early development (lin-4) in addition to being highly conserved across species (let-7) [15, 16]. MiRNAs are small (19–23 nucleotides), single-stranded noncoding RNA molecules involved in posttranscriptional control of genetic expression of a large number of genes, acting as regulators of mRNAs degradation and/or blocking protein translation of distinctive parts of target proteins via binding to their corresponding sequences within the 3′‐untranslated regions (3′UTRs) of target mRNAs [17–19]. It has been manifested that miRNAs, particularly serum miRNAs, are stable in circulation because they are resistant to RNase's digestion. This shows great promise in becoming a novel diagnostic biomarker of MetS and its components [20]. The primary miRNAs are transcribed by the microprocessor complex, consisting of the RNAse III-like enzyme Drosha and its dsRNA-binding partner DGCR8, into precursor miRNAs. With the help of Exportin-5, the pre-miRNAs are transported to the cytoplasm. There are two main pathways; in the canonical pathway, the pre-miRNA is cleaved in the cytoplasm by the RNAse III-like enzyme Dicer in collaboration with the cofactors TRBP and PACT [21]. In addition to these canonical miRNA processing pathways, some alternative mechanisms have recently been revealed. MiR-451's maturation is processed in a Dicer-independent, but AGO2-dependent way [21, 22].

Given the high morbidity of MetS and its components, as well as the noninvasive and stable role of miRNAs on diagnosis, here we will make an overall review on the association between the main components of MetS, IR, obesity, diabetes, lipid metabolism, hypertension, hyperuricemia, stress, and miRNA and its targeting proteins or relevant pathways. This review can help us make a further understanding on the promising application of miRNAs for early diagnosis and treatment of MetS and its components.

## 2. IR and miRNAs

**Figure 1:**
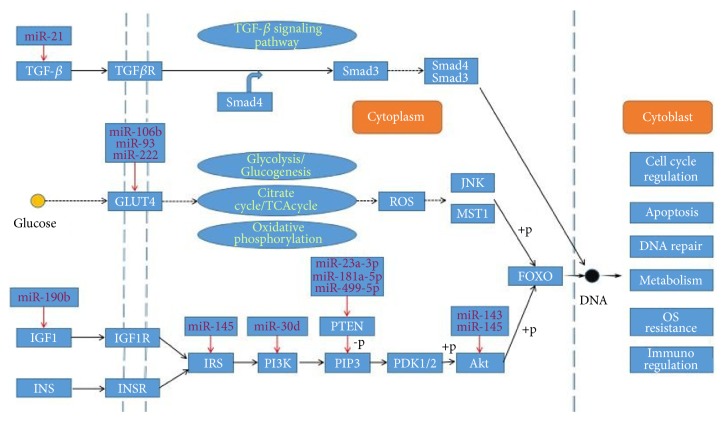
The red arrowhead represents inhibition. ① miR-499-5p, miR-181a-5p, and miR-23a-3p: the overexpression of miR-499-5p can directly downregulate PTEN, which can inhibit the PI3K/AKt pathway. As a result, they can improve the IR. ② miR-143: the overexpression of miR-143 can inhibit the insulin-induced Akt activation to cause an IR. ③ miR-145: miR-145 can inhibit the phosphorylation of IRS-1 and Akt. ④ miR-190b: miR-190b can decrease the level of IGF-1. ⑤ miR-21: miR-21 can inhibit the TGF-*β*1/Smad3 pathway. ⑥ miR-222 and miR-93, miR-106b: inhibit the GLUT4. ⑦ miR-30d: inhibits PI3K.

IR has been identified as a reduced response of target tissues (skeletal muscle (SM), liver, and adipocytes) to insulin [23]. As a result, excess glucose fails to be sufficiently absorbed by cells even in the presence of insulin, thereby causing an increase in the level of blood glucose. In a previous study, magnetic resonance spectroscopy (MRS) was applied to study the etiology of IR. It has been demonstrated that almost all of the IR in T2DM patients can be attributed to insulin-stimulated muscle glycogen synthesis defects, resulting from deficiency of insulin-stimulated glucose transport and phosphorylation activity. Compared with the absolute quantity of fat in the body, the distribution of fat is more important for inducing IR. Because obesity causes fat to accumulate not only in adipocytes, but also in muscle and hepatocytes, leading to IR in these organs [24], in recent years, miRNAs have appeared as novel biomarkers for diagnosis of quite a few diseases. Nevertheless, the relationship between miRNAs and IR remains obscure. Here we will make a review on the possible link between IR and miRNAs. And the roles of some miRNAs acting on the metabolic pathway are described in Figure 1.

### 2.1. In Livers

In an experiment, mice were treated with a high fructose diet to induce IR; it has been shown that there were several miRNAs overexpressed such as miR-19b-3p, miR-101a-3p, miR-30a-5p, miR-582-3p, and miR-378a-3p, whereas miR-223-3p, miR-33-5p, miR-128-3p, miR-125b-5p, and miR-145a-3p were downregulated. The potential target genes of these miRNAs include IRS-1, FOXO-1, SREBP-1c, SREBP-2, SREBP-2, Insig-1, and Insig-2 (Ing-2a and -2b), which are involved in insulin signaling (IRS-1 and FOXO-1), the synthesis of hepatic lipids (SREBP-1c, SREBP-2, ChREBP, Insig-1, and Insig-2 (insig-2a and -2b)), as well as MTTP and apoB that are involved in VLDL assembly and secretion. It has been illustrated that a miRNA can be involved in multiple pathways, and a gene may be a collective target of several miRNAs [25]. In a study conducted by Lei Wang et al., they have tested that in db/db mice's livers the downregulated miR-499-5p impaired the insulin signaling pathway and glycogen synthesis pathway while upregulated miR-499-5p promoted the insulin signaling pathway and glycogen synthesis. Double luciferase report assay and western blot analysis showed that phosphatase and tensin homolog deleted on chromosome 10 (PTEN) was the target gene of miR-499-5p. Moreover, the level of miR-499-5P has been proved to be positively related to the PI3K/Akt /GSK signaling pathway and glycogen synthesis through directly inhibiting PTEN [26]. However, miR-499-5p can be bound with PTENP1, which is a pseudogene located at 9p13.3 and shares high similarity with PTEN. As a result, high expression of PTENP1 can induce IR [27]. In another IR model with obesity, miR-143 specifically inhibited insulin signaling at the level of Akt activation in livers, but not in SM, whereas upstream receptor signaling remained intact [28]. Similarly, overexpression of miR-145, which could be upregulated by resistin both in vivo and in vitro, could inhibit glucose uptake and contribute to IR through decreasing phosphorylation of Akt and IRS-1 in HepG2 cells [29]. A previous study has reported that development of hepatocellular carcinoma (HCC) was accompanied by an conspicuous reduction in serum IGF-1 levels and an overexpression of miR-190b. IGF-1 is generated mainly in livers and plays a crucial role in promoting growth and regulating metabolism, and low level of IGF-1 may induce IR. Overexpression of miR-190b in patients with HCC decreases the expression of IGF-1, whereas inhibition of miR-190b resulted in upregulation of IGF-1. So it has been demonstrated that IGF-1 was a direct and functional target of miR-190b, which has been identified consecutively by luciferase reporter assay, qRT-PCR, western blotting, and immunofluorescence analysis. It was discovered that low serum IGF-1 levels were associated with IR and poor overall survival in HCC patients [30]. In a study conducted by Qian Xu et al., they found that in NAFLD patients, miR-103 was overexpressed in the serum. MiR-103 was positively correlated with HOMA-IR, TG, and BMI, respectively, and miR-103, TG, and BMI were all independent factors associated with HOMA-IR. Moreover, miR-103, TG, BMI, and HOMA-IR were all risk factors for NAFLD. High expression of miR-103 led to IR by downregulating caveolin-1, which is a target gene of miR-103 and a critical regulator of the insulin receptor. In addition, silencing of miR-103 decreased total fat content by reducing adipocyte size [31].

### 2.2. In SM

SM exerts a crucial role in regulating whole body glucose homeostasis, which is responsible for 70%–80% of insulin-stimulated glucose uptake. It has been reported that high expression of miR-676 could significantly upregulate fatty acid oxidation–related proteins, along with downregulating complement activation– and acute-phase response–related proteins. Furthermore, hepatic ectodysplasin A (EDA), together with its intronic miRNA, miR-676, was upregulated and released from hepatocytes under conditions of obesity and could act on SM as its main target tissue; its increased expression in liver occurs due to a systemic IR. The probable mechanism was that EDA-A2 could enhance c-Jun N-terminal kinase (JNK) phosphorylation and inhibit phosphorylation of Ser307 of IRS-1 in vivo to cause obesity-induced IR [32]. Overexpression of miR-16 has been reported to inhibit the synthesis of insulin-stimulated SM protein, while several other miRNAs involved in muscle metabolism may be responsive to IR, including miR-149, -182, and -696, deserving further investigation [33]. In another study, rats with intrauterine growth restriction have high expressions of miR-29a, which has a direct interaction with its target gene, peroxisome proliferator-activated receptor *δ* (PPAR*δ*), and PPAR*δ*'s coactivator, peroxisome proliferator-activated receptor- *γ* coactivator-1 *α* (PGC-1 *α*). MiR-29a could suppress the PPAR *δ* /PGC-1 *α* -dependent signals. Overexpression of miR-29a also caused a decreasing level of glucose transporter 4 (GLUT4), the most important glucose transporter in SM, which partially decreases insulin-dependent glucose uptake. Taken together, they induce SM IR (SMIR) [34]. There are certain drugs targeting the miRNAs associated with IR in SM. One of the most widely used hypoglycemic drugs, metformin, has been demonstrated that it can improve IR by reducing expression of miR-21 in a concentration-dependent manner. It has been proven that the expression of miR-21 is closely related to SM IR, through the TGF- *β* 1/Smad3 pathway [23]. In contrast, thiazolidinediones work by increasing insulin sensitivity by acting on PPAR *δ* specifically in the adipocyte, resulting in a redistribution of fat away from the hepatocyte and myocyte and into the adipocyte to improve IR [24].

### 2.3. In AT

Omental AT (OAT) exerts a central role in IR in gestational diabetes mellitus (GDM). In a study exploring the differential expression of miRNAs in OAT from GDM patients, miR-222 is upregulated in OAT from GDM patients and negatively correlated with the expression of ERa protein and GLUT4 protein. miR-222 has been reported to be upregulated in the diabetic rats AT and to regulate estradiol concentrations of PCOS patients characterized by intrinsic IR and dysfunctional glucose metabolism in AT [35]. It has been reported that 60-70% of patients with PCOS suffer from IR. A study has identified that only GLUT4 was downregulated in PCOS women with IR in the IRS/PI3- K/Akt pathway signaling. It has been shown that the decrease of GLUT4 is targeted by miR-93 [36]. TNF-*α* also plays a pivotal role in IR. Several miRNAs are thought to be involved in the development of AT IR. For example, miR-181a-5p and miR-23a-3p have been reported to negatively correlate to adiposity and HOMA-IR. Both miRNAs function to upregulate insulin-stimulated Akt activation. When the two miRNAs were inhibited either individually or in combination, PTEN and S6K protein expression increased, predicting that miR-181a-5p and miR-23a-3p could prevent TNF-*α* -induced IR in AT through regulation of PTEN and S6K expression [37].

### 2.4. Other miRNAs in IR

It has been previously known that chronic pistachio consumption significantly improves glucose and insulin metabolism as well as other cardiovascular risk factors in the prediabetes state [38]. In an experiment, after consuming a pistachio-supplemented diet (PD) for 4 months, subjects without T2DM had significant lower circulating levels of miR-192 and miR-375 and the changes in expression of miR-15a, miR-21, miR-126, and miR-29b were significantly increased, whereas miR-21 had no significant change. Levels of circulating miR-192 and miR-375 were positively correlated with plasma glucose, insulin, and HOMA-IR, indicating that the increasing levels of miR-192 and miR-375 are two risk factors of IR. It can be indicated that miR-15a, miR-21, miR-29b, and miR-126 are negatively correlated with IR [39]. Chronic stress may facilitate the development of metabolic disorders including IR and T2DM. Si-Si Wang et al. have proved that stress-associated miRNAs such as miR-18a and miR-34c were, respectively, independent positive and negative predictors of HOMA-IR [40]. MiR-106b, miR-27a, and miR-30d were significantly elevated in rats with IR. It has been found that miR-106b, miR-27a, and miR-30d critically regulate the main components (GLUT4, MAPK 14, and PI3K regulatory subunit *β*) of the GLUT4 signaling pathway by binding with the 3'-UTRs of Slc2a4 (encoding GLUT4), Mapk14 (encoding mitogen-activated protein kinase (MAPK) 14), and Pik3r2 (encoding PI3K regulatory subunit beta) genes, which respectively carry the binding sites for miR-106b, miR-27a, and miR-30d [41].

## 3. Obesity and miRNAs

The spread of obesity has prevailed all over the world whether in developed or developing countries. Obesity is the biggest risk factor in a series of metabolic diseases, including certain diabetes, CVDs, and cancers, and is correlated with a wide cluster of metabolic disorders including inflammation and IR. The significant rise in obesity has put serious stress on public healthcare networks across the globe [42, 43]. In particular, central obesity, also called abdominal obesity, which means the fat is accumulated in visceral AT (VAT), plays a pivotal role in obesity-related disorders. Metabolism in obese individuals is associated with changes in miRNAs expression profiling. It has been confirmed that most of the miRNAs are downexpressed while most of proteins are overexpressed in VAT from the obese subjects [44, 45].

MiR-33 has been demonstrated to cause a rapid expansion of fat mass in mice. When the miR-33 was knocked out, the mice suffered from a rapid expansion of fat mass. What is more, the mice with miR-33 knocked out had an increase in food intake, lipid accumulation in the liver, and higher levels of both HDL-C and total cholesterol after being fed with a high fat diet. It has been indicated that even on a control diet, mice lacking miR-33 would develop IR in several key metabolic tissues [42]. Dicer is an important miRNA processing endoribonuclease on the processing of miRNAs. In an experiment, knocking out Dicer in proopiomelanocortin (POMC) precursor-expressing cells in arcuate nucleus of the hypothalamus (ARC) has led to increased food intake, hyperglycemia, impaired glucose tolerance, and secondary obesity. This is because ARC can secrete neuropeptides agouti-related protein and neuropeptide Y which are orexigenic as well as POMC precursor which is anorexigenic. It has shown that miRNAs can regulate pathophysiological procedures not only via binding with the 3-UTR of the targeting mRNAs, but by changing the expression of Dicer [46]. It is of interest that studies on epigenetics have been a hot issue in recent decades. It is the characteristics of inheritable and reversible chemical changes in the genome other than those in the DNA sequence. The epigenome is responsible for nuclear information, regulating development, tissue differentiation and cellular responsiveness. Epigenetic information is controlled by genome sequence, environmental exposure, and stochasticity. DNA methylation is one of the three forms of epigenetic information, a covalent modification of the nucleotide cytosine at the 5'site, which is generally associated with gene silencing in all unicellular and multicellular organisms [47, 48]. A study conducted by M. L. Mansego et al. has found that DNA methylation levels of three selected CpG sites respectively located in miRNA coding regions (miR-1203, -412, and -216A) were associated with obese children [49].

The relationship between obesity and metabolic diseases remains unclear, but growing evidence has implicated that obesity-induced inflammation is an important mechanism linking obesity to the MetS in metabolically active tissues, especially in white AT. The mechanisms involved are complex and seem to be mediated by inflammation which triggers downstream signals that appear to affect metabolism including lipids. As such, obesity has been demonstrated to be a chronic low-grade inflammatory disease state that results in IR, T2DM, and CVD [45, 50]. Obesity during pregnancy has been reported to generate a long-term effect on the health of the offspring including risk of developing the MetS. The offspring from the obese mothers had decreased cytokine (TNF-*α*) and chemokine (CCl2 and CCL7), which were closely associated with an inflammation reaction in their AT. This was accompanied by downexpression of miR-706, which could directly regulate translation of the inflammatory proteins IL-33 and calcium/calmodulin-dependent protein kinase 1D. Programming of miRNAs in AT may be a contributing mechanism of regulating expression of inflammatory signaling molecules [51].

What is more, AT can regulate metabolism through the secretion of adipokines. It has been shown that most of the circulating exosomal miRNAs, which serve as a previously undescribed form of adipokine, are generated from adipocytes [52]. Additionally, Leptin and IL-6a are both common adipokines that are involved in inflammation. Adiponectin can improve insulin sensitivity, vasodilation, and lipid oxidation, while protecting against atherogenesis. Perrine Goguet-Rubio et al. has conducted a study which aimed to build a panel to detect abnormal levels of cytokines and several miRNAs including 197-3p, miR-320a 23-3p, 221-3p, 27a-3p, and 130a-3p in patients with MetS. These miRNAs had been demonstrated to correspond to metabolic diseases. In this study, participants were divided into three groups: Control, Obese, and MetS. The result showed that among the three groups, leptin, leptin to adiponectin ratio, and IL-6 levels were highest in MetS, and levels in Control were lowest. Adiponectin and miRNAs levels were both highest in Control, but lowest in MetS. The indexes in obesity were all between MetS and Control [4]. Xi-Mei Zhang et al. have identified 23 active miRNA-transcription factors (TF)-gene regulatory pathways that were significantly related to obesity-related inflammation. In these pathways, 6 adipokines, including IL-1 *β*, CCL2, RBP4, VEGFA, SERPINE4, and TNF- *α* were involved. It has been suggested that specific miRNAs may play a significant role in regulating inflammation in human circulation through their effects on adipokines levels, which may be mediated by indirect effects on TF circuits. miRNAs could therefore constitute a novel potential therapeutic target for obesity and its comorbidities, especially the MetS [53]. MiR-335 is associated with inflammation in obesity, through regulating the expression of TNF-*α*. Polyphenol-rich green tea (GT) extract has proved to improve AT metabolism and ameliorating IR and inflammation by inhibiting miR-335 expression. GT prevents weight gain and reverses almost all obesity-induced metabolic complications in mouse white AT, as it is associated with an increase in energy consumption and an improvement in AT inflammation and IR-associated gene expression, which was closely linked to lower expression of miR-335 [50].

## 4. Diabetes and miRNAs

DM is one of the most common metabolic diseases globally characterized by IR and pancreatic *β* cell dysfunction [54]. Obesity is considered as a lifestyle-associated and heritable disorder, as well as a major risk factor for secondary diseases like T2DM. Obesity and T2DM are the result of the interaction of genetic, epigenetic, and environmental factors. MiRNAs plays a crucial role in this physiopathologic process [55]. It has also been commonly reported that type 1 diabetes mellitus (T1DM) traditionally had a low BMI and microangiopathic complications; however, macroangiopathy and the MetS were also exceptional. Though the incidence of microangiopathy can be reduced by improving glycemia, it can result in a higher rate of severe hypoglycemia and weight gain. The components of the MetS and IR have been associated with chronic T1DM complications, and cardiovascular disease is now the major cause of death in these patients [56].

There are some new technologies to explore the mechanisms of miRNAs on DM. Next generation sequencing (NGS) has been demonstrated to be used to identify novel isoforms of miRNA and analyze miRNomes in other tissues. In an experiment using NGS, it has identified that there were 20 miRNAs from subcutaneous AT (SAT) samples and visceral AT that differed between obese (O) and normal-weight (N) individuals. Hsa-miR-146b-3p, hsa-miR-146b-5p, hsa-miR-223-3p, hsa-miR-450b-5p, and hsa-miR-22-3p were significantly upregulated while hsa-miR-205-5p was deeply downregulated in SAT-O. The expression of only one miRNA, hsa-miR-424-3p, was significantly upregulated in the visceral AT of obese subjects (VAT-O) when compared to VAT-N [57]. A study used a novel computational framework (miRNAs-quantitative trait loci-Scan) to identify miRNAs that contribute to obesity and T2DM. Manipulation of miR-31 in human Simpson-Golabi-Behmel syndrome adipocytes regulated the expression of GLUT4, PPAR *γ*, IRS1, and acetyl-CoA carboxylase *α*. MiR-15b levels were correlated to baseline blood glucose concentrations and might be a predictive biomarker for diabetes. This study can help enhance the ability of discovering miRNAs as regulative elements [55]. Growing evidence has shown that single nucleotide polymorphisms (SNPs) within miRNA genes or miRNA-binding sites can change the relationship between miRNAs and their target mRNAs and thus regulate target gene expression. These kinds of SNPs are known to contribute to susceptibility to a variety of diseases, including T2DM. The CDKN2A and CDKN2B gene encode two kinase inhibitors, p16INK4a and p15INK4b, respectively, which suppress two regulators of pancreatic b-cell replication. Xiaojing Wang et al. selected and genotyped three miR-binding SNPs of CDKN2A/B gene (rs1063192, rs3217992, and rs3088440) in 839 cases of GDM and 900 controls. The CC genotype of CDKN2B rs1063192 in the hsa-miR-323b-5p binding site increased the risk of GDM in pregnant Chinese Han women [58]. Later, they found another two miR-binding SNPs SLC30A8 rs2466293 and INSR rs1366600 increased GDM susceptibility. These two studies have proved that miR-binding SNPs were a novel source of GDM susceptibility loci [59].

It has been shown that miR-92a could inhibit apoptosis induced by a high-glucose (HG) environment and increase the insulin secretion and proliferation by targeting KLF2. Furthermore miR-92a could inhibit the HG-induced caspase3 activity and decrease its expression to escape apoptosis. What is more, miRNA-92a could attenuate OS through decreasing the generation of reactive oxygen species [54]. HG in GDM not only induces the OS in the mothers, but exerts a similar effect on their offspring, even embryos. A study has found that miR-27a was upregulated in embryos exposed to diabetes along with high glucose in a dose- and time-dependent manner. The overexpression of miR-27a can inhibit the nuclear factor-erythroid 2-related factor 2 which controls antioxidant enzymes including the glutamate-cysteine ligase catalytic subunit, glutamate-cysteine ligase modifier subunit, and glutathione S-transferase A1, inducing OS in the embryos [60]. It has been demonstrated that miRNA-340 was upregulated in GDM via decreasing its target gene, PAIP1. It has been also indicated that the expression of miRNA-340 responded similarly to different glucose and insulin concentrations [61].

As is referred above, the relationship between T1DM and MetS should gain more emphasis. MiRNAs as biomarkers for implicating the risk of T1DM have also become more and more significant. MiR-375 is upexpressed in the endocrine pancreas, whereas it has been found to be downregulated in the serum of T1DM children and the downexpression of miR-375 might be a general biomarker of metabolic alterations and inflammation associated with the disease [62]. In another trial, a miRNA -based model was used to the training dataset with high diagnostic accuracy for T1DM. This model was based on six candidates differentially expressed miRNAs identified in recent-onset T1DM, including miR-642a-3p, miR-320c, miR-1225-5p, let-7b-5p, miR-26b-5p, and miR-144-3p. Levels of miR-1225-5p and miR-320c were significantly increased in streptozotocin-induced b-cell damage mice and INS-1 cells prior to glucose elevation in the progress of diabetes in nonobese diabetic mice. These two miRNAs may become promising and early diagnostic markers on T1DM [63]. Though there is no significant difference between individuals with high risk of T1DM and healthy controls on the expression of miRNAs, miRNA expression in the T1DM group clearly differs from high-risk individuals and healthy controls. This might be due to the major metabolic disorders around the time of diagnosis. Only miR-497-5p and miR-339-3p have been shown to be respectively downregulated (p = 0.034) and upregulated (p = 0.043) in high-risk group compared to healthy group. Meanwhile, the expression of C-peptide was positively correlated with miR-106b-3p, serum glucose concentration was negatively correlated with miR-151a-3p, -766-3p, -146b-5p and -5p, and the level of HbA1c was positively associated with several miRNAs, including miR-140-3p, -148a-3p, -23a-3p, -222-3p, -29a-3p, and let-7b-3p [64].

Diabetes is significantly associated with atherosclerosis, causing severe complications. Endothelial cells (ECs) are the primary targets of glucose-induced cellular damage in chronic diabetic complications due to their innate ability to uptake glucose independent of insulin activity. Endothelin-1 (ET-1) is known to be pathogenetically significant in several chronic diabetic complications. A study has proved that miR-1 could prevent the HG-induced ET-1 upregulation and target fibronectin (FN) which is mediated partly by ET-1 [65]. In the context of diabetes-associated atherosclerosis (DAA), previous reports indicate that serum let-7 levels are lower in patients with CVD and T2DM. The overexpression of let-7 can suppress mediators of vascular inflammation, including IL-6, IL-1*β*, and NF-*κ* B, and regulate SMC and ECs activation and inflammation, via regulation of PDGF and TNF-*α* signaling. So let-7 can act as an important target to prevent DAA [66]. MiR-24 performs a significant function in regulating ECs function and the TGF-*β* signaling pathway through targeting the gene FURIN. Circulating levels of miR-24 are significantly decreased in T2DM patients with coronary heart diseases (CHD). The level of YKL-40 in serum is associated with the CHD and myocardial infarction and increase in TIDM and T2DM patients, as a high risk for the development of CVD and IR. MiR-24 has been demonstrated to be significantly and negatively correlated with YKL-40 in either T2DM patients with CHD or CHD patients [67]. Moreover, a study has investigated the correlation between serum miR-217 and the severity of diabetic kidney disease (DKD) determined by albuminuria and patients with T2DM (all of them were newly diagnosed DKD) were divided into three groups: normoalbuminuric group, microalbuminuric group, and macroalbuminuric group. It showed that circulating miR-217 levels were significantly increased in T2DM patients compared with healthy controls and positively related to the level of albuminuria. It can be indicated that miR-217 may be involved in the development of DKD by promoting chronic inflammation, renal fibrosis, and angiogenesis [68].

## 5. Lipid Metabolism and miRNA

Emerging as pivotal regulating factors of cholesterol metabolism and promising therapeutic targets for treating cardiometabolic disorders, miRNAs have played an increasing role in regulating lipid metabolism associated disorders including MetS, obesity, and atherosclerosis in recent years [69]. There is a growing interest in the therapeutic role of miRNA inhibitors and miRNA mimics on treating lipid metabolic disorders. Because the biological pathways regulated by miRNAs might be conserved, making them valuable therapeutic targets [70]. Argonaute crosslinking and immunoprecipitation demonstrated that snoRNA host gene 16 (SNHG16) heavily binds AGO and has 27 AGO/miRNA target sites along its length, indicating that NHG16 may act as a competing endogenous RNA (ceRNA) to “sponge” miRNAs off their cognate targets. SNHG16 has been demonstrated to be positively associated with Stearoyl-CoA Desaturase (SCD), which is involved in fatty acid catabolism [71].

MiR-122, the most abundant miRNA in the livers (occupies approximately 70%), has a crucial role in liver development and function that together with hepatocyte nuclear factor which appears to maintain the hepatic cell phenotype and its inhibition can decrease total serum cholesterol. The modulation of cholesterol metabolism is one of the most studied biological processes since its first isolation from gallstones in 1784 [70, 72, 73]. According to genetic loss-of-function and gain-of-function experiments, the orphan nuclear receptor REV-ERBa is a major circadian regulator of mir-122 transcription in liver. This result well matches with the circadian control of metabolic regulation. This circadian rhythm has been demonstrated to inhibit miR-122 expression via antisense oligonucleotide (ASO) strategy which resulted in the up- and downregulation of hundreds of mRNAs [74]. A study explored the role of the liver-specific miR-122 in the adult liver through inhibiting it in mice with a 2'-O-methoxyethyl phosphorothioate ASO. This study has shown that miR-122 inhibition in normal mice caused a decrease in plasma cholesterol levels, hepatic fatty acid and cholesterol synthesis rates, along with an increase in hepatic fatty acid oxidation. In a diet-induced obesity mouse model, miR-122 inhibition resulted in decreased plasma cholesterol levels and a significant improvement in liver steatosis, as well as reductions in certain lipogenic genes [75]. A study was conducted in 62 morbidly obese (MO), 30 moderately obese (ModO), and 8 normal-weight controls, and their BMIs were respectively < 25kg/m2, BMI 32 – 38 kg/m2, and > 40 kg/m2. The result has shown that hepatic miR-122 expression was decreased in the MO group when compared to ModO and MO patients with NASH showed higher miR-122 circulating levels than MO with simple steatosis [76]. Women with GDM can change the lipid metabolism and certain pathways in their offsprings in a sex-dependent manner. It has been identified that TC and TG in the livers of male fetuses of GDM rats were increased compared to controls and female fetuses of GDM rats; however, there were significant decreases of TC, TG, phospholipids; and free fatty acids in the livers of female fetuses of GDM rats compared to controls and male fetuses of GDM rats. Further molecular identification has shown that PPAR*γ* was only increased in the liver of male fetuses of GDM rats, along with a decrease of miR-130, a miRNA targets PPAR*γ*. Respectively, PPAR*δ* was found to be upregulated paralleled with a reduction of miR-9, targeting PPAR*δ* in the liver of female fetuses of GDM alone [77]. What is more, miR122 plays a pivotal role in the development of hepatitis virus such as maintaining the stability of hepatitis C virus, whereas inhibiting the expression and replication of hepatitis B virus by a miR-122-cylin G1/p53-HBV enhancer regulatory pathway [73].

Besides miR-122, miR-33 also plays a major role in regulating lipid metabolism as an intronic microRNA located with the SREBP-2 gene, regulates cholesterol efflux, fatty acid *β*-oxidation, and HDL metabolism [72]. Complications of atherosclerosis are the most common causes of death in western societies. According to epidemiologic studies, increased levels of circulating cholesterol are significantly important in regulating the development of atherosclerosis both in human and experimental animals [78]. MiR-33 has been demonstrated to play a crucial role in this program by many studies [69–81]. One of the possible mechanisms is the lipid accumulation and inflammation in macrophages. Nathan L. et al. have identified this mechanism through knocking out the miR-33 in mice models. According to their study, macrophage-specific miR-33 ablation engendered dramatic reduction in the accumulation of cholesterol esters, atherosclerotic plaque size, and lipid content [79]. Conversely, it has been observed by CARS microscopy that miR-33 drives lipid droplet accumulation in macrophages. Autophagy promotes the degradation of cytoplasmic components in lysosomes and plays a pivotal role in the catabolism of stored lipids to maintain cellular homeostasis. MiR-33 can inhibit the autophagy to increase cholesterol accumulation and induce atherosclerosis [80]. miR-33 has been also proven to regulate macrophage inflammation so reducing miR-33 can ameliorate plaque inflammation [81].

In other living things, miRNAs also play significant roles in their lipid metabolism. Lipid bodies are the main source of nutrients in mycobacterium tuberculosis (Mtb). It has been identified that Mtb could escape the autophagy, lysosomal function, and fatty acid oxidation to support bacterial replication through inducing miR-33 and its passenger strand miR-33*∗*'s expressions [82]. Aedes aegypti mosquitoes need an army of energy to meet the need for their reproduction. Lipids are the major energy storage. Using the CRISPR-Cas9 system to disrupt miR-277 (targeting insulin-like peptides 7 and 8) led to failures in both lipid storage and ovary development [83]. In Bacteria-challenged* Apostichopus japonicus*, miR-31 has been demonstrated to disrupt lipid metabolism homeostasis, thus resulting in cell apoptosis by targeting complement C1q tumor necrosis factor-related protein 9 and disturbing ceramide pathways [84]. Leishmaniasis patients are usually observed to have an abnormal lipid metabolism. A study has shown that liver-specific miR-122 levels in the Leishmaniasis murine model were reduced; the possible mechanism may be that* Leishmania *surface glycoprotein gp63, a Zn-metalloprotease, targets Dicer1 to prevent miRNA ribonucleoprotein (miRNP) complex formation. As a result, the expression of lipid metabolic genes was altered, causing a reduced level of serum cholesterol and increased liver parasite burden [85]. Therefore, it can be a promising therapeutic strategy to use miRNAs to treat relevant pathogens infection.

## 6. Hypertension and miRNAs

Essential hypertension (EH) has been a major health burden and the most probable risk factor for certain CVD, such as myocardial infarction, stroke, heart failure, and renal disease. MiRNAs have become major therapeutic targets for CVD in an era of developing precision medicine [86–88]. Interestingly, a study has proved that ablation of DGCR8 can cause a downexpression of a majority of miRNAs and attenuate multiple signaling pathways including ERK1/2 and Akt. As a result, blood pressure and vascular reactivity were significantly decreased. The possible mechanism might be the inhibition of cell proliferation, migration, and neointima formation [89]. But the accurate pathway including associated genes, mRNAs, and miRNAs remains elusive. In order to explore the relationship between MetS and its components, Dwi Setyowati Karolina et al. conducted a study of 5 groups, which were respectively healthy controls, MetS, T2DM, hypercholesterolemia (HCL), and hypertension (HPT). The result has shown that the expression of miR-103, -17, -183, -197, -215, -23a, -335, -509-5p,-584, -652, and -765 was observed to be specific to MetS and HCL subgroups. Dysregulation of miR-130a, -195, and -92a was unique to MetS and HPT. MiR-150, -192, -27a, -320a, and -375 levels were found to be upregulated in both MetS and T2DM while showing the opposite trend in HCL and HPT. From this data, it has been confirmed that MetS developed from a cluster of these risk factors [90]. Sibao Yang et al. have demonstrated that the SNP-modified posttranscriptional gene regulation by miRNAs could be a potential pathogenetic mechanism of EH via a study conducted on activating transcription factor1 (ATF1) rs11169571 polymorphism. They have identified that ablation of miR-1283 in human aorta vascular smooth muscle cells could enhance the expression of ATF1 mRNA as well as the radical oxygen species (ROSs) [86]. Similarly, Mohsen Ghanbari has systematically investigated the association of genetic polymorphisms in the seed regions of miRNAs with cardiometabolic phenotypes. They found that rs2168518: G>A, a seed region variant of miR-4513, was correlated with fasting glucose, LDL-cholesterol and total cholesterol, systolic and diastolic blood pressure and risk of coronary artery disease. Further experiments identified that GOSR2 was a target of miR-4513 and demonstrated that rs2168518 can change the regulation of this gene [91]. MiR-145 has been identified to play an important role in the development of certain CVD. In a previous study, it has been demonstrated that miR-145 was upregulated in atherosclerotic plaques from hypertensive patients compared to controls, while treatment with angiotensin receptor blockers may increase miR-145 levels, although this data did not garner statistical significance probably due to the limited sample size [92]. But it failed to illustrate the idiographic molecular mechanism.

Later, a study was conducted on spontaneously hypertensive rats (SHR) and rat vascular endothelial cells (RVECs) aiming to investigate the role of miR-145 on hypertension. In the SHR group, it can be observed that there was an upregulation of miR-145 and a significant decrease in nitric oxide (NO) content. In RVECs, inhibited miR-145 caused a significant overexpression of solute carrier family 7 member 1 (SLC7A1) and phosphorylated endothelial nitric oxide synthase. SLC7A1 is an amino acid transporter that regulates arginine metabolism, which is closely associated with endothelial function and NO synthesis. It has been indicated that miR-145 can regulate the pathogenesis of hypertension by targeting SLC7A1 [93]. Aerobic exercise training (ET) can reduce blood pressure and improve outcomes in CVD. MiR-16, miR-21, and miR-126 have been proven to exert a significant role in VEGF, which is important for the development of hypertension. In a study conducted by Tiago Fernandes et al., ET can decrease blood pressure by downregulating miRNA-16 and -21 levels and elevating VEGF and Bcl-2 levels and restore soleus endothelial NO synthase levels. MiR-126 level downregulated in SHRs paralleled with an increase of phosphoinositol-3 kinase (PI3K) but normalized in SHRs with ET. It has been shown that ET promoted peripheral revascularization in hypertension, which could be associated with certain miRNAs. But they did not study whether there was any direct targeting relationship between the pairs of miRNAs and genes [94]. Using luciferase assay, reverse transcription-quantitative polymerase chain reaction, and western blot analyses, miR-31a-5p has been demonstrated to inhibit the activity of TP53 to decrease the expression of p53, a tumor suppressor protein that is involved in the development of restenosis and atherosclerosis, vascular smooth muscle cells (VSMCs) growth and cell death. It has been shown that miR-31a-5p can regulate the program of hypertension through promoting the proliferation of arterial SMCs and inhibiting the apoptosis by directly targeting TP53 [95]. Endothelial dysfunction is closely associated with the pathogenesis of hypertension [96]. In recent years, using loss- and gain-of-function in vitro or in vivo methods, several studies have identified that various miRNAs that predominate in ECs have a significant effect on modulating normal ECs functions, including proliferation, apoptosis, and migration, which are crucial for the regulation of normal vascular processes [97]. MiRNAs in microvascular endothelial cells (MECs) serve a pivotal role on regulating hypertension. Alison J. Kriegel et al. have found that miR-26a-5p, -92a-3p, -92b-3p, -181a-5p, -181b-5p, miR-21-5p, -30a-5p, -98, -125a-5p, -125b-5p, -181a-5p, -92a-3p, -92b-3p, -27a the let-7 family of miRNAs, including let-7a, let-7e, let-7f, and let-7g in MECs downregulated the expression of certain hypertension Genome-wide linkage and association studies genes including adrenomedullin, plasma membrane calcium-transporting ATPase 1, FURIN, fibroblast growth factor 5, Golgi SNAP receptor complex member 2, jagged 1, SH2B adapter protein 3, and T-box 3. What is more, adrenergic receptors A1b, A2A, and A2B were suppressed respectively by miR-22-3p, -30a-5p, and -30e-5p. Adrenergic receptor B1 was inhibited by let-7b, let-7c, let-7e, and let-7g and endothelin receptor B by miR-92a-3p and miR-92b-3p [98].

OS is thought to be a risk factor of hypertension. The mechanism is that OS can increase the expression of ROSs, including nicotinamide adenine nucleotide phosphate oxidases and cyclooxygenases (COXs), which can reduce NO signaling in the endothelium and interfere with endothelial function. Douglas F. Dluzen et al. conducted a study on African American and white females who were either normotensive or hypertensive and have demonstrated that the upexpression of miR-103a-2-5p or miR-585-5p can affect DNA damage and cell survival by targeting poly-(ADP-ribose) polymerase 1, a DNA damnification sensor protein involved in DNA repair and other cellular processes [88]. Overproduction of reactive oxygen species generated in mitochondria (mt) has been implicated as a risk factor of hypertensive cardiomyopathy. A study has identified that mi-21 can reduce blood pressure by targeting mtDNA-encoded cytochrome b (mt-Cytb). In this study, it can be observed that the level of miR-21 in SH patients was significantly higher compared to controls. What is more, through transfecting miRNA mimic into rats' circulation via tail vein, mt-Cytb level was increased, which resulted in ameliorating the blood pressure [99]. Angiotensin II (AngII) is a key molecular in the regulation of blood pressure by MAPK signaling pathway. In order to identify the potential therapeutic targets of AngII-induced hypertension, Xiaoli Wu et al. used gene expression profiles of GSE93579 and GSE75815 to demonstrate differentially expressed genes between AngII-induced hypertension and control samples based on meta-analysis. The result showed that miR-124a might be involved in the pathogenesis of hypertension via targeting Ebf3 and Rgs7bp, which possibly serves as a novel and effective strategy for the treatment of hypertension [96].

## 7. Hyperuricemia and miRNAs

Hyperuricemia tends to cause gout and nephrolithiasis, and it is an independent risk factor for CVD and chronic kidney disease (CKD), in addition to hypertension, diabetes, and obesity [100, 101]. The uric acid (UA) level has been demonstrated to be positively related to male sex, waist circumference, abnormal levels of TGs, TC, blood glucose, obesity, sedentariness, night-time SBP, and birth weight [101, 102]. Fructose-induced increase of UA is a risk factor of MetS [103, 104]. By contrast, it has been reported that lowering the UA level can postpone the development of CKD in people with diabetes [105]. UA is generated from the metabolism of purines, and its overexpression has been proved to exert a key role in human diseases [104, 106].

A study aiming to investigate the relationship between high concentrations of uric acid (HUA) and angiogenesis has found that miR-92a-Krüppel- like factor 2 (KLF2)-vascular endothelial growth factor-A (VEGFA) axis plays an important role in the development of UA-associated cardiovascular injury. Under the stimulation of HUA, miR-92a downregulation increased KLF2 expression, subsequently inhibiting VEGFA, which inhibited angiogenesis. This study reveals a possible mechanism for cardiovascular injury induced by hyperuricemia [100]. Inflammasome activation by UA crystallization and superoxide free radicals generated by xanthine oxidase (XO) have been reported to be closely related to the inflammation induced by UA, and the XO inhibitors, such as allopurinol, oxypurinol, febuxostat, and topiroxostat, can reduce serum UA level and improve OS. Febuxostat has been used to ameliorate atherosclerosis, nonalcoholic steatohepatitis, diabetic renal injury, hypertension, dyslipidemia, and IR [103]. When it comes to the relationship between UA and cardiometabolic risk factors, a study has evaluated the anthropometric parameters, office and 24-hour blood pressure measurements and metabolic indexes, including HDL-C, TG, insulin, HOMA index and UA in overweight and moderate obese youth. The number of abnormal metabolic risk factors has been proven to be positively correlated with UA level [102]. In cardiometabolism, inflammation has been proven to exert a pivotal role in cardiac metabolic disorder associated with hyperuricemia. Rosangela Spiga et al. have identified the effect of UA on the expression of inflammatory biomarkers in human hepatoma HepG2 cells and the signaling pathway on which UA acts. The result has shown that UA stimulates the expression of C-reactive protein, fibrinogen, ferritin, and complement C3 in a dose-dependent way but can be reversed by benzbromarone, a specific inhibitor of UA transporters. These in vivo and in vitro data suggested that hyperuricemia might promote the expression of hepatic inflammatory factors by activating the proinflammatory NF- *κ*B signaling pathway. This UA-induced inflammation in hepatic cells helps us to understand the similar mechanism in cardiometabolism [107]. In another study, it has been shown that IL-6 and IL-8 were positively related to the degree of urate deposits as well as the miR-155 levels [108]. But no association between IL-6, IL-8, and miR-155 has been found. The SNP rs1333049 (C/G) was found to be correlated with CAD. A study was to evaluate the relationship between this SNP and gout pathogenesis. The result has shown that rs1333049 genotypic and CC allelic frequencies were positively related to the increased risk of gout and this SNP is homologous to miR-519 and miR-520 [101].

## 8. Stress and miRNAs

Chronic stress can exert a harmful effect on health such as MetS and its components through complex interactions among neuroendocrine systems and energy homoeostasis, playing a significant role in gene expression via epigenetic regulation. One of the major neuroendocrine mechanisms is the response of hypothalamus–pituitary–adrenal (HPA) axis, in which glucocorticoid secretion is the final hormonal effector [40, 109–112]. miRNAs have been identified to exert an important role in the functioning of the central nervous system and as a novel therapeutic strategy of chronic psychological stress such as anxiety and mood disorders as well as in preclinical models of psychological stress [113–115].

When exposed to chronic stress, some people respond positively to stress, whereas others respond negatively to stress and develop anxiety as well as depression. The vulnerability to stress is regulated by a redesigned neurovascular unit characterized by increased neural activity, vascular remodeling, and proinflammatory mechanisms in the ventral hippocampus (vHPC), targeted by miR-455-3p and miR-30e-3p. In the passive coping rats, miR-30e-3p was upregulated while miR-455-3p was downregulated. Moreover, the proinflammatory cytokine VEGF-164 can increase vulnerability to stress, while the nonsteroidal anti-inflammatory drug meloxicam can improve vulnerability [116]. Mice exposed to chronic ultramild stress suffered from depression-like behaviors and a downexpression of the brain-enriched miR-124 in hippocampus. What is more, miR-124 exerts an important role in susceptibility/resilience to chronic stress by targeting histone deacetylase 4,5, and glycogen synthase kinase 3 *β* [113]. In another study, overexpression of miR-17-92 in hippocampal neural progenitors has been shown to exert a crucial effect on neurogenesis and anxiety- and depression-related behaviors as a response to chronic stress in mice. Further molecular investigation has shown that miR-17-92 affects neurogenesis by regulating the glucocorticoid pathway, especially serum- and glucocorticoid-inducible protein kinase-1(Sgk1), resulting in rescuing proliferation dysfunction induced by corticosterone [115]. Under a short-period mild restraint stress, it has been found that adiponectin, C1Q and collagen domain containing gene (Adipoq) and prolactin receptor mRNAs in the cerebellum were upregulated. Stress also regulates the expression of miR-186 and miR-709 in hippocampus and prefrontal cortex; moreover, miR-186 targets the gene epidermal growth factor receptor (EGFR) pathway substrate 15 gene, which is an endogenous substrate for the EGF1 receptor kinase. It has been suggested that miRNA's regulation on gene expression plays an important role in stress response and related neural function [117]. Similarly, miR-18a-5p,-34a-5p, -135a-5p, -195-5p, -320-3p, -674-3p, -872-5p have been identified to be upregulated in the ventral tegmental area of stressed rats, making the rats more resistant to stress than anhedonic animals [118]. A study has identified that miR-20b, -21, -26b -16, and -134 in saliva were closely associated with stress when treated with Trier Social Stress Test to induce acute psychological stress [112]. It has been reported that glucocorticoids can inhibit the expression of CRF type 1 receptor (CRF-R1) mRNA and protein. miR-449a has been proven to exert a crucial effect in this stress-induced, glucocorticoid-mediated downregulation of CRF-R1 expression by binding to the 3'-UTR of CRF-R1 mRNA [110]. The norepinephrine transporter (NET) and glucocorticoid receptors play important roles in responding to stress. NET mRNA and protein levels in rats are regulated by chronic stress and by administration of corticosterone, which is mediated through glucocorticoid receptors. It has been demonstrated that overexpression of miR-181a and miR-29b can directly target the NET and glucocorticoid receptors, which may be related to stress-induced upregulation of the noradrenergic phenotype [111].

MiR-335 has been proven to mediate cell cycle arrest as a stress response to DNA damage via balancing the activities of the retinoblastoma (Rb) and p53 tumor suppressor pathways. miR-335 can activate the p53 tumor suppressor pathway after altering Rb1 (pRb/p105) levels [119]. The miR-34 and miR-449 families share the same seed sequence, being described to mediate antiproliferation and to be involved in cellular differentiation. Natural killer cell immunoreceptor ligand ULBP2 (also named NKG2DL) surface expression is closely associated with cellular stress such as infection, heat shock, and DNA damage. ULBP2 is sensitive to the early identification and clearance of cytotoxic lymphocytes (CTL) and also provides a natural barrier against tumor development. It has been reported that miR-34a and miR-34c can directly target ULBP2 mRNA; miR-34a is negatively correlated with expression of ULBP2 surface molecules. Meanwhile, the antioncogene p53 can activate miR- 34a and miR-34c under the condition of small molecule inhibitor Nutlin-3a, resulting in the inhibition of ULBP2 [120]. miR-34 has also been reported to be an important molecular in the development of metabolic stress-associated metabolic diseases, such as lipid and glucose metabolism [40, 121]. Overexpression of miR-34a or downexpression of hepatocyte nuclear factor 4*α* (HNF4*α*) in the liver can improve the development of atherosclerosis in patients with nonalcoholic steatohepatitis, diabetic mice and mice fed a HFD [121]. Chronic stress is also a dependent risk factor of IR and Cortisol is positively associated with glucose, HOMA-IR, and waist circumference. A study has shown that expression levels of miR-18a and miR-34c were significantly correlated with cortisol, corticotropin-releasing factor (CRF), and interleukin 6 (IL-6). Moreover, the expression levels of miR-18a and miR-34c were positively and negatively correlated with risk of T2DM and IFG, respectively [40]. Besides, upregulation of let-7b, miR-144, and miR-29a and downregulation of miR-142 were significantly correlated with T2DM, IFG, and IR. What is more, these miRNAs were significantly associated with stress hormone levels [109]. In a study aiming to investigate the stress-associated miR-21/miR-21*∗*'s function on the development of metabolic liver disorders, Calo et al. have applied HFD on the miR-21/miR-21*∗* knockout mice. Under the condition of HDF stress, glucose intolerance, steatosis, and adiposity, they were ameliorated in miR-21/miR-21*∗* knockout mice. The mechanism is that ablation of miR-21/miR-21*∗* led to increased insulin sensitivity and regulated the expression of multiple crucial metabolic transcription factors involved in the procedure of lipid and glucose metabolism [122]. In another study, heavy drinkers (HD, defined as regular alcohol consumption over the past year of at least 8 standard drinks/week for women and at least 15 standard drinks/week for men) have shown a substantial expression of miR-10a and miR-21 after suffering from psychological stress. 79 genes were identified to express differently in the HD group after the exposure to stress. Many of these genes, which form part of the TAR-RNA binding protein (TRBP)-associated complex, are positively regulated by miR-10a and miR-21. Meanwhile, ACTH' concentration was closely associated with level of miR21, but not miR-10a. The expressions of miR-10a, miR-21, and several of their target genes can be altered to respond to acute psychological stress [123]. Calmodulin binding transcription activators (CAMTAs), mainly consisting of CAMTA1 and CAMTA2, are involved in cell differentiation in response to environmental cues and play a pivotal role as common integrators to stress responses. The overexpression of CGCG-core motifs in the promoter of the miR-212/miR-132 cluster has been demonstrated to upregulate insulin secretion. Under the stimulation of high glucose, it has shown that CAMTA1 and CAMTA2 can directly target these motifs to increase the insulin secretion, with the coactivation of Nkx2-2, an essential islet transcription factor [124].

Chronic inflammatory stress has been suggested as a crucial risk factor for the development of atherosclerosis. During this procedure, macrophages and VSMCs swallow a large number of lipoprotein particles and become foam cells. The upregulation of adenosine triphosphate (ATP)-binding membrane cassette transporters A1 (ABCA1) and G1 (ABCG1) can promote cholesterol efflux. A study has demonstrated that inflammatory cytokines (such as IL-6 and TNF- *α*) or miR-33a-5P can increase intracellular lipid accumulation and decrease apoA-I-mediated cholesterol efflux by downregulating ABCA1 and ABCG1 in the absence or presence of LDL in macrophages. On the contrary, increasing level of anti- miR-33a-5P neutralized the effects of inflammatory cytokines. It can be suggested that inflammatory cytokines may inhibit ABCA1/ABCG1-mediated cholesterol efflux by upregulating miR-33a-5P [125]. ROSs may react with macromolecules of cells like fatty acids, DNAs, proteins, causing damage to these macromolecules [126]. OS occurs when oxidant production in cells and plasma exceeds antioxidant activity, which mainly accounts for two factors: one is that energy surplus such as high glucose or high FFA leads to overproduction of hydrogen peroxide and superoxide ion in mitochondria; another reason is the activation of NADPH oxidase by AngII receptor. It has been shown that OS can downregulate insulin-dependent stimulation of insulin signaling elements and glucose transport activity via activating the serine kinase p38 mitogen-activated protein kinase (p38 MAPK) pathway in SM, resulting in IR and T2DM [127]. OS is the common procedure of major causative factor for major depression caused by inflammation, autoimmune tissue damage, and chronic psychological stress. A study aiming to explore the relationship between OS and depression has found that malondialdehyde (MDA), which belongs to oxidant parameters, was found to be significantly upregulated in depressed patients compared with controls, whereas the antioxidant defense biomarkers ascorbic acid and superoxide dismutase (SOD) were significantly decreased. Moreover, nitrite and ceruloplasmin were decreased but these results did not reach statistical significance. It can be indicated that oxidative stress plays a pivotal role in the development of depression [126]. In a study conducted on 200 patients with oral cancer, it has shown that more than a half of the subjects have concomitant MetS. Subjects who had an areca-nut-chewing habit suffered from higher levels of OS, inflammatory parameters, and fasting glucose than those who never chewed, as well as a significantly lower HDL-C level and SOD activity. Moreover, the MS components were positively correlated with OS and inflammation especially that of hyperglycemia for the reason that areca nut and its nitrosamines might promote glucose intolerance [128]. Areca-nut-chewing induced MetS may be due to OS and inflammation. Another study has demonstrated that autonomic dysfunction led to an increase in both inflammation and OS probably by inducing hemodynamic and metabolic dysfunctions observed in MetS, to promote cardiometabolic disorders [129]. Overexpression of miR-4673 has been proved to promote paclitaxel-induced apoptosis, mitochondrial membrane potential loss, and ROS generation by directly targeting 8-oxoguanine-DNA glycosylase-1 [130]. Overexpression of ROS has been proved to induce dysfunction, proinflammatory, and apoptotic death of ECs. Neural progenitor cells exosomes can improve the OS and dysfunction in ECs through targeting Nox2 and VEGFR2 pathways by miR-210 [131]. It has been reported that OS can activate the liver fibrogenic cells (myofibroblasts) to upregulate fibrosis-related genes, leading to hepatic fibrogenesis. MiR-706, a liver-enriched miRNA, has been found to inhibit OS-induced hepatic fibrogenesis by directly targeting PKC*α* and TAOK1 [132]. Hepatic miR-192-5p has been found to be significantly downregulated in OS-induced acute liver injury, being dependent on stimulation with TNF. This change can improve the hepatic function under the liver injury, via targeting Zeb2 [133]. The overexpression of miR- 200-3p can promote OS-induced cell death via mimicking p38a deficiency. MiR-200-3p has been proven to be upregulated by phosphorylated p53, which is conversely phosphorylated by p38a [134].

## 9. Conclusion and Prospective

We made an overview on the recent findings made on the relevant miRNAs and their target proteins or associated pathways of IR, obesity, diabetes, lipid metabolism, hypertension, hyperuricemia, and stress. Liver, SM, AT, and endothelium cells are the four main targeted tissues of the IR and other components' development. We reviewed the demonstrated miRNAs in liver, SM, AT, and other miRNAs associated with IR, such as miR-19b-3p, miR-101a-3p, miR-30a-5p, miR-378a-3p, miR-582-3p, miR-223-3p, miR-33-5p, miR-145a-3p, miR-125b-5p and miR-128-3p, miR-499-5p, miR-143, miR-190b. Moreover, GLUT4 is the most common molecule and its relevant pathways play a pivotal role in the progress of IR. There are many other miRNAs involved in regulating the relevant genes and pathways of main components of MetS, which can help find out the potential diagnostic and therapeutic miRNAs for MetS. In recent years, miRNAs mimics and ablation have been widely used for the association study and therapy. ASO is an effective and novel method for miRNAs inhibition. Besides IR, proinflammation and stress (especially OS) have been demonstrated to exert crucial roles in metabolic disorders. However, the role of HAP-axis stress related miRNAs on metabolism has not been fully explored. With the increasing chronic psychological stress in modern society as well as the high morbidity of MetS, it is of utmost importance to focus on further associated investigation into the role of HAP-axis stress related miRNAs in MetS.
